# Annual meeting of the EpiGeneSys Network of Excellence – Advancing epigenetics towards systems biology

**DOI:** 10.1002/bies.201500015

**Published:** 2015-03-16

**Authors:** Jon Houseley, Caroline S. Hill, Peter J. Rugg‐Gunn

**Affiliations:** ^1^ Epigenetics ProgrammeThe Babraham InstituteCambridgeUK; ^2^ Laboratory of Developmental SignallingCancer Research UK London Research InstituteLondonUK

**Keywords:** epigenetics, epigenotype, modelling, systems biology

## Abstract

The third annual meeting of the EpiGeneSys network brought together epigenetics and systems biologists to report on collaborative projects that apply quantitative approaches to understanding complex epigenetic processes. The figure shown represents one meeting highlight, which was the unexpected emergence of genotype versus epigenotype in control of cell state.

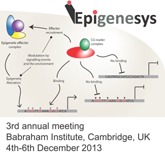

## Introduction

The European Commission network of excellence “EpiGeneSys” has been in operation since 2010 and the third annual meeting took place at the Babraham Institute in Cambridge from 4th to 6th December 2013. EpiGeneSys is an ambitious project studying epigenetics with an emphasis on systems biology. The marriage of these two disciplines is desirable because systems biology approaches are essentially designed to make sense of biological systems that are highly dynamic and involve a plethora of components, both of which are features of epigenetics.

In addition to the 22 permanent members of EpiGeneSys, there are 20 RISE1 (research integrating epigenetics and systems biology) members, who are early career stage researchers, and 97 associates. The meeting in Babraham gave this illustrious group of scientists and their lab members the opportunity to come together for a few days of excellent talks and discussions to catch up on the scientific highlights of the network, as well as the network's other functions such as training, career development and public engagement.

The research of EpiGeneSys is divided into four main areas covering dynamics of epigenetic regulators, the relationship between genotype and epigenotype, the influences of environment and nutrition on the epigenome and finally, building an integrated computational epigenetics framework. At the Babraham meeting there were highlights from all of these areas, and in this report we present a selection to illustrate key issues under discussion and to indicate the future trajectory of EpiGeneSys research.

## Genotype to epigenotype

A central theme of the meeting was the extent to which genotype can influence epigenotype. Adrian Bird (Wellcome Trust Centre for Cell Biology, Edinburgh, UK) suggested that we should consider CpG islands in the context of “CG signaling” rather than focusing on CpG methylation. Bird challenged the audience to think about a reader of CG signalling, CFP1, as a sequence‐specific DNA binding protein, even though CG is clearly a very short sequence, that is able to influence epigenetic modifications by recruiting histone‐modifying machinery. Examination of artificial DNA insertions showed that altering GC‐content and CpG density of an ectopic locus is sufficient to establish different epigenetic states 1. These findings support the proposal that DNA sequence and transcription factor binding determine the epigenetic landscape, which in turn reinforces the active or repressive state.

Evolutionary diversity can also be used effectively to explore the relationship between DNA sequence and epigenetic landscape. Axel Imhof (Ludwig Maximilians University of Munich, Germany) presented work about the molecular basis for species divergence 2. Genetic and biochemical assays showed that the interaction of two genes, hybrid male rescue (*Hmr*) from *Drosophila melanogaster* and lethal hybrid rescue (*Lhr*) from *Drosophila simulans*, causes hybrid incompatibility due to mislocalisation of an HMR/LHR complex throughout the genome, altered regulation of transposable elements and impaired cell proliferation. Levels of HMR and LHR are different between the two *Drosophila* species, which may have arisen as a response to species‐specific changes in the abundance of transposable elements that occurred during divergent evolution.

The relationship between genetics and epigenetics is not all one‐way however; Daniel Rico (CNIO, Madrid, Spain) described how the timing of DNA replication can define the rate at which copy number variants arise. He demonstrated that replication timing reflects the evolutionary age of duplicated genes, and that the late replicating regions of genomes tend to be newer. Replication timing, an almost completely epigenetic property, allows fast and slow evolving regions to be defined. Consistently, primate‐ and rodent‐specific genes tend to be in late replicating regions, suggesting that this conserved epigenetic property controls the rate of genetic change and evolutionary innovation 3.

Connections between underlying genomic features and the epigenome are also revealed by genome‐wide analyses of chromatin states. Guillaume Filion (CRG, Barcelona, Spain) has previously used computational classification strategies to interrogate combinations of proteins and epigenetic marks, and reported that five major types of chromatin can be distinguished in *Drosophila* cells. New data presented at the meeting showed that the same five chromatin types are also observed in human K562 cells, revealing that many common features at the genome‐wide level are conserved between *Drosophila* and human cells. Filion extended these studies by performing the same analysis for human embryonic stem cells (ESC), and although broadly similar, several interesting differences were observed. “Black” chromatin, for example, which is characterised by the absence of DNA binding factors, is detected with a similar abundance between K562 and ESC, yet shows relatively poor overlap in its location within the genome. These findings remind us that chromatin state must be defined by more than DNA sequence alone.

There are situations where chromatin state, however, can be affected by genetic changes depending on genome position. Gael Yvert (CNRS, Lyon, France) described experiments comparing histone acetylation patterns in three different yeast strains. Nucleosome positions and acetylation patterns varied in complex ways; sites with conserved nucleosome positioning and conserved acetylation were observed, but sites showing conservation of neither or only one of these properties were also readily detected. To elucidate genetic and epigenetic contributions to this heterogeneity, histone acetylation was stripped by drug treatment and then allowed to re‐establish over a number of generations. Quantitative differences in levels of histone modifications were found to be either “labile” or “persistent” depending on whether they were re‐established or not after drug treatment. Remarkably, “persistent” variations correlated well with those linked to genetic determinants whereas “labile” variations did not, showing clear contributions of both genetic and epigenetic information to intra‐species variation of the histone modifications landscape 4.

The influence of the genome on the epigenome is of more than theoretical importance. Cihangir Yandim (Imperial College London, UK) reported the results of a study on the autosomal recessive disorder Friedreich's Ataxia, which is caused by an expansion of GAA triplet repeats in an intron of the *FXN* gene. The expanded triplet repeat tract seeds an aberrant region of heterochromatin, which spreads bidirectionally, leading to transcriptional silencing of the *FXN* gene. Importantly, heterochromatinisation could be inhibited using the histone deacetylase inhibitor nicotinamide. Administration of nicotinamide to a cohort of Friedreich's Ataxia patients in a Phase II clinical trial led to a significant increase in *FXN* mRNA and protein levels, proving the concept that drugs targeting epigenetics can have clinical applications 5.

## New insights into dosage regulation

Dosage compensation in various species has long provided a paradigm for epigenetic regulation of gene expression. Asifa Akhtar (Max Planck Institute of Immunobiology and Epigenetics, Freiburg, Germany) showed that two related MOF‐containing complexes, MSL and NSL, can control dosage compensation in mouse ESC, but achieve it through two distinct pathways 6. MSL binds the two active X‐chromosomes in female ESC and reinforces *Tsix* transcription, thereby limiting *Xist* expression. In contrast, NSL positively regulates pluripotency factors, including *Oct4* and *Sox2*, in turn leading to suppression of *Xist* expression. These findings reinforce the concept that multiple mechanisms are used to ensure correct dosage regulation during development.

Caroline Dean (John Innes Centre, Norwich, UK) addressed how environmental signals can influence gene expression and phenotype. An interesting epigenetic system allows environmental signals perceived at one stage to be “remembered” until later in development. Vernalisation, the promotion of flowering by cold, involves gradual PcG‐mediated epigenetic silencing of floral repressor FLC in *Arabidopsis thaliana*. But the important question now addressed by the Dean group is how this process is reset every generation? They screened for mutants impaired in the epigenetic reprogramming of FLC. In one hypomorphic mutant, FLC is switched on, but fails to reach wild‐type levels. The mutated gene was identified as *Elf6*, encoding a jumonji C domain protein likely to be an H3K27me2/3 demethylase. H3K27me3 levels remain elevated in mutant seeds, therefore demethylase activity is required to erase H3K27me3 in the resetting between generations 7.

## The role of non‐coding RNAs in genome function

Numerous studies have shown that epigenetic marks can be deposited by non‐protein coding RNAs (ncRNAs), particularly those involved in transcriptional repression and heterochromatin formation. This has been well studied at fission yeast centromeres, where ncRNAs from the outer centromeric repeats are processed by the RNA interference (RNAi) machinery to form siRNAs. These siRNAs direct the formation of centromeric heterochromatin, but the connection between the RNAi machinery and the chromatin modifying enzymes only became clear with the recent discovery of the connector protein Stc1. Elizabeth Bayne (Institute of Cell Biology, Edinburgh, UK) reported a structural analysis of Stc1, showing that the zinc finger domain interacts directly with Argonaute while the C‐terminal interacts with the CLRC silencing complex, providing the molecular basis of the connection between RNAi and heterochromatin formation 8.

How the RNAi machinery selects targets for processing into siRNA and hence defines heterochromatin domains has remained somewhat mysterious, as many genomic regions produce sense and antisense RNAs that could technically form RNAi substrates. Using an RNAi system reconstituted in budding yeast, Jon Houseley (Babraham Institute, Cambridge, UK) demonstrated that transcripts from high‐copy genomic regions are inherently better at forming double‐stranded RNA than those from single copy regions. This unexpected influence of genome sequence on an apparently epigenetic process provides a simple answer to an old problem, as selective silencing of high‐copy regions has long been thought to require a mechanism by which the cell can count DNA copies. To be effective however, this RNAi‐mediated silencing mechanism would require the whole genome to be transcribed, providing a potential function for recently discovered pervasive transcription of eukaryotic genomes 9.

Mammalian cells also use ncRNAs to control transcriptional silencing. In this area, Claire Rougeulle (Université Paris Diderot, Paris, France) described recent work from her laboratory regarding the control of X‐inactivation in human ESC. X‐chromosome inactivation in mammalian cells is orchestrated by the ncRNA *XIST*, but human ESC express an apparently antagonistic ncRNA, *XACT*, which coats the active X‐chromosome. X‐inactivation is variable in human ESC compared to mouse, potentially reflecting a balance between *XIST* and *XACT* expression, and here it was shown that *XACT* expression precedes *XIST* loss and X‐reactivation, and that *XACT* is present on both X‐chromosomes in embryos when both Xs are active.

## Epigenomics in single cells

Single cell sequencing approaches have the potential to revolutionise epigenetics. Heather Lee (Babraham Institute) revealed that even apparently homogenous ESC cultures in fact show distinct epigenetic heterogeneity. However, some cell populations are indeed highly homogenous; Sebastien Smallwood (Babraham Institute) presented the first genome‐wide methylomes from single cells, in this case from oocytes, which showed very little sample‐to‐sample variability providing an important validation of single cell methodologies 10.

John Marioni (European Bioinformatics Institute, Cambridge, UK) described how single cell expression profiling of brain cells from the marine annelid *Platynereis*, combined with in situ profiling for selected marker genes, allowed reconstruction of the expression profile of the brain with single cell resolution. Expanding such techniques to more complex organisms is highly challenging. However, Andreas Ladurner (Ludwig Maximilians University of Munich) presented surprisingly effective approaches for profiling selected neuronal cell populations in *Drosophila*. By expressing tagged ribosomes, RNA polymerases or histones using specific GAL4 driver lines, it was possible to measure gene expression and chromatin structure changes in specific sets of neurons, glia and adipocytes within the fly head. This was used to demonstrate how gene expression changes when a fly is fed normally or fasted.

## Perspectives

**Figure 1:**
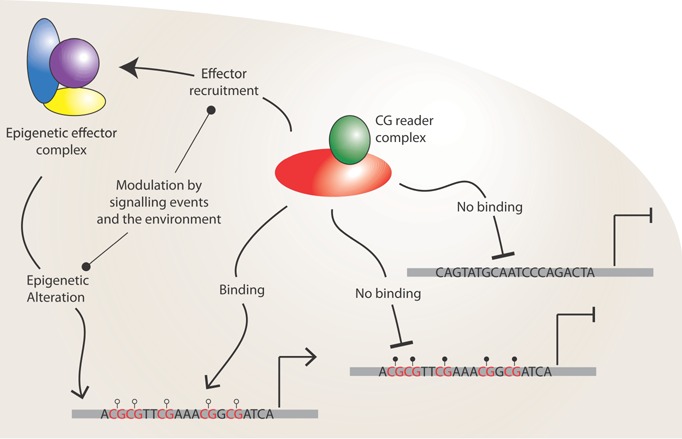
Epigenetic readers as sequence‐specific binding proteins. Representation of a sequence‐specific reader protein binding to unmethylated CpG (open circles), but not to methylated CpG (filled circles) or to sequences without CpG. The reader recruits other epigenetic modifiers to define the epigenetic status of the sequence. This process can be modulated at various steps by extrinsic and intrinsic signalling activities, adding flexibility to the response. Alteration of local epigenetic states impacts the functional state of the surrounding genomic regions, controlling the transcription of neighbouring genes for example.

It was exciting to see the emergence of an unexpected theme at this meeting: the influence of DNA sequence on the epigenome. This concept unites many of the core research areas of EpiGeneSys and was a common discussion point during the coffee breaks. Overall, a model emerges whereby epigenetic processes coordinate nuclear function and transcriptional states, but on one level these states can be defined by DNA sequence elements and their associated binding proteins. Of course, external signalling events require changes in the transcriptional output of the genome and trigger changes in epigenetic profiles, however, these profiles are constrained by underlying sequence considerations (Fig. 1). Hence, the epigenome functions to establish and maintain transcriptional states that to some extent can be defined at the level of the DNA sequence. These exciting and emerging concepts will be an active area of future work for members of EpiGeneSys.
